# Adult lifespan effects on functional specialization along the hippocampal long axis

**DOI:** 10.3389/fcogn.2026.1767179

**Published:** 2026-05-05

**Authors:** Caitlin R. Bowman, Cara I. Charles, Saisha M. Birr

**Affiliations:** Department of Psychological and Brain Sciences, University of Wisconsin-Milwaukee, Milwaukee, WI, United States

**Keywords:** episodic memory, fMRI, functional connectivity, healthy aging, hippocampus, lifespan

## Abstract

**Introduction:**

There has been increasing attention to differences in function along the hippocampal long axis, with the posterior hippocampus proposed to have more variable signals that are well-suited to representing idiosyncratic details in memory, and the anterior hippocampus having less dynamic signals that are well-suited to integration. Whether long axis functional specialization persists into older age is not well-understood, despite known age-related declines in the level of detail in memories.

**Methods:**

We used a large database of resting state fMRI data (*n* = 337 humans of both sexes included) from across the adult lifespan (ages 18–88) to determine the degree of functional differentiation across the hippocampal posterior-anterior axis. Our first approach was to measure the correlation of signals within hippocampal subregions. Our second approach was to measure functional connectivity between hippocampal subregions and the rest of the brain. For both approaches, we tested how well functional differences along the hippocampal long axis accounted for individual and age differences in episodic memory.

**Results:**

Within the hippocampus, we found a more positive age slope (i.e., increasing similarity of signals) for the most posterior hippocampal region compared to the intermediate and anterior region, consistent with the posterior hippocampus losing some of its heterogeneous signaling in older age. Patterns of whole-brain connectivity showed significant differences in the age trajectories between hippocampal subregions for a number of target regions, including several frontal connections. Yet we did not find strong evidence that either within-hippocampal signals or differences in functional connectivity were associated with age-related episodic memory decline.

**Conclusion:**

Age differences in hippocampal long axis functional organization were apparent during rest but were limited in how well they accounted for memory decline.

## 1 Introduction

The hippocampus is critical for episodic memory function (Scoville and Milner, 1957), and it shows structural and functional decline in healthy (Hu and Li, 2020; Leal and Yassa, 2015; O'Shea et al., 2016) and pathological aging (Anand and Dhikav, 2012; Peng et al., 2014). A functional differentiation along the hippocampal long axis has been proposed based primarily on work in animals and healthy young humans (Poppenk et al., 2013), with the posterior hippocampus proposed to represent fine-grained details, and the anterior hippocampus proposed to have coarser representations that integrate across larger windows of time and space. It is well-known that episodic memory declines in healthy aging, especially for the kinds of fine-grained details that are proposed to be represented in the posterior hippocampus (Hashtroudi et al., 1989; Old and Naveh-Benjamin, 2008; Prull et al., 2006; Toner et al., 2009). Yet the extent to which older age disrupts the hippocampal long axis functional gradient has not been well-established.

Early work in rodents focused on differences in spatial processing along the hippocampal long axis, with lesions to rodent dorsal hippocampus (analog of human posterior hippocampus) causing greater disruption to spatial learning than equally large lesions to the ventral hippocampus (analog of human anterior hippocampus; Moser et al., 1993). Later work demonstrated that the whole hippocampus was involved in representing spatial information, but that place cells in the ventral hippocampus represented much larger areas of space than those in the dorsal hippocampus (Kjelstrup et al., 2008), such that they would not be detected in many studies working on a smaller scale. These differences in the spatial function of the hippocampus were also complemented by other forms of long axis differences (Strange et al., 2014), including differences in gene expression (Thompson et al., 2008), connectivity with different locations within the entorhinal cortex (Dolorfo and Amaral, 1998), and studies showing impairment to memory tasks requiring generalization (transitive inference) with ventral hippocampal lesions (Bunsey and Eichenbaum, 1996). Thus, work in young animals indicated that hippocampal long axis specialization likely arises from multiple underlying properties of the hippocampus and affects multiple processing domains.

One proposal of the hippocampal long axis specialization is that differentiation emerges due to differences in the granularity of signals within the hippocampus (Poppenk et al., 2013). It is proposed that the posterior hippocampus is well-suited to represent more fine-grained details because its signals are more variable, allowing it to capture changes that happen over short periods of time or that vary across small areas of space. The anterior hippocampus, in contrast, is proposed to have more stable signals, making it well-suited to integrating across time and space. This hypothesis was supported by an influential study in young adult humans using fMRI data (Brunec et al., 2018). The authors correlated the fMRI timeseries across voxels within hippocampal subregions that spanned the posterior-to-anterior axis. This approach, where the timeseries from each voxel in a region is correlated with the timeseries of every other voxel in the region is known as intervoxel similarity (IVS), with higher values indicating greater consistency of the signals within a region. Brunec et al. (2018) showed that fMRI timeseries were less correlated among voxels in the posterior hippocampus compared to those within the anterior hippocampus, consistent with the putative role of the posterior hippocampus in representing details. This pattern emerged both in resting-state data and in task-based spatial navigation data, with links between behavioral measures of spatial navigation and self-reported time spent in episodic remembering and simulation during the resting state data (Brunec et al., 2018). Although this study has been highly influential, its results have not been consistently replicated. A later study used the same intervoxel similarity approach with a larger sample size and resting state data, finding a different pattern: signals were least similar to one another in intermediate portions of the hippocampus and more similar in the furthest anterior and posterior portions (Thorp et al., 2022). However, this study did not test the extent to which within-hippocampus signals across subregions were related to behavior, leaving an open question as to whether this resting state pattern has implications for memory performance. Thus, there remains some question of the nature of within-hippocampal signals across the hippocampal long axis even in young adults.

Work testing age-related differences in intrahippocampal signals has been limited. Nonetheless, a prior study used resting-state fMRI data from 198 individuals aged 30–85 years old to measure the similarity of signals within the medial temporal lobe (MTL; including but not limited to the hippocampus; Salami et al., 2016). They also measured longitudinal change in within-MTL signal similarity over an approximately 5-year period. Rather than the IVS measured described above, they used temporal concatenation independent components analysis (ICA) to empirically derive spatial clusters with similar timeseries. Across the sample, they identified separate posterior and anterior MTL clusters. Further, they showed longitudinal increases in the similarity of signals within the posterior MTL cluster, whereas signals within the anterior MTL cluster became less similar to one another starting at approximately aged 60. Further, increases in the similarity of posterior MTL signals were associated with poorer episodic memory abilities (Salami et al., 2016). Thus, these findings provide initial evidence that disruptions to within-MTL signals contribute to age-related declines in episodic memory, with the posterior MTL playing a particularly important role. However, because these analyses included both neocortical MTL and the hippocampus, it remains unclear the extent to which findings were driven by differences along the hippocampal long axis vs. extrahippocampal MTL.

In young animals and humans, patterns of structural and functional connectivity between the hippocampus and the rest of the brain are also known to contribute to hippocampal long axis specialization (Catenoix et al., 2011; Duvernoy et al., 2005; Fanselow and Dong, 2010; Frank et al., 2019; Jung et al., 1994; Kahn et al., 2008). Prior work in healthy young adults has shown that parts of lateral prefrontal cortex and lateral parietal cortex have stronger functional connectivity with the posterior than the anterior hippocampus, while the ventromedial prefrontal cortex showed greater coupling with the anterior compared to posterior hippocampus (Frank et al., 2019). Other work has positioned the hippocampal functional gradient within larger medial temporal lobe systems (Libby et al., 2012; Ranganath and Ritchey, 2012; Rugg and Vilberg, 2013). According to this framework, the posterior hippocampus is part of the posterior medial system that includes the parahippocampal cortex, retrosplenial cortex, posterior cingulate, precuneus, angular gyrus, and anterior thalamus. The anterior hippocampus is part of the anterior temporal system, which includes the perirhinal cortex, lateral orbitofrontal cortex, amygdala, and temporal pole. In older adults, separable posterior medial and anterior temporal networks have been detected (Das et al., 2015), and healthy aging has been associated with weaker within-network connections for both the posterior medial and anterior temporal networks (Hrybouski et al., 2023). A prior study that included older adults in a sample that was predominantly children, young, and middle aged adults showed age-related decreases in connectivity between the posterior hippocampus and the anterior cingulate and medial superior frontal gyrus (Panitz et al., 2021). Yet the extent to which these effects were driven by differences in advanced age and their relationship to memory abilities remains unclear.

In the present study, we tested the degree to which patterns of hippocampal functional specialization persist across the adult lifespan and how any differences in hippocampal specialization relate to known age-related declines in episodic memory (Fraundorf et al., 2019; Old and Naveh-Benjamin, 2008; Spencer and Raz, 1995; Toner et al., 2009). We used resting-state fMRI data from individuals aged 18–88 (*n* = 337) from the Cambridge Center for Aging and Neuroscience (CamCAN) dataset (Shafto et al., 2014). We compared signals across three anatomically defined segments along the hippocampal posterior-anterior axis (tail, body, head) in terms of both the similarity of signals within each hippocampal subregion and functional connectivity between each subregion and the rest of the brain. We related these metrics to age-related differences in performance on an episodic memory task. Based on the proposed role of the posterior hippocampus in representing fine-grained details in memory and known age-related decline in episodic memory, we expected signals within the posterior hippocampus to become more similar to one another in older age, making it less able to represent fine-grained details. We also expected to find greater age-related differences in whole-brain functional connectivity of the posterior hippocampus relative to the anterior hippocampus. Lastly, we expected differences in the functional properties of the posterior hippocampus to at least partially explain age-related declines in episodic memory performance.

## 2 Methods

### 2.1 Data and code availability

The raw data come from a publicly available dataset (https://www.cam-can.org). Intervoxel similarity values (IVS), functional connectivity values, scores on the behavioral tasks used, and nuisance covariates for individual subjects are publicly available through the Open Science Framework (https://osf.io/qba8n/). Analytic code is also available.

### 2.2 Participants

**Figure 1:**
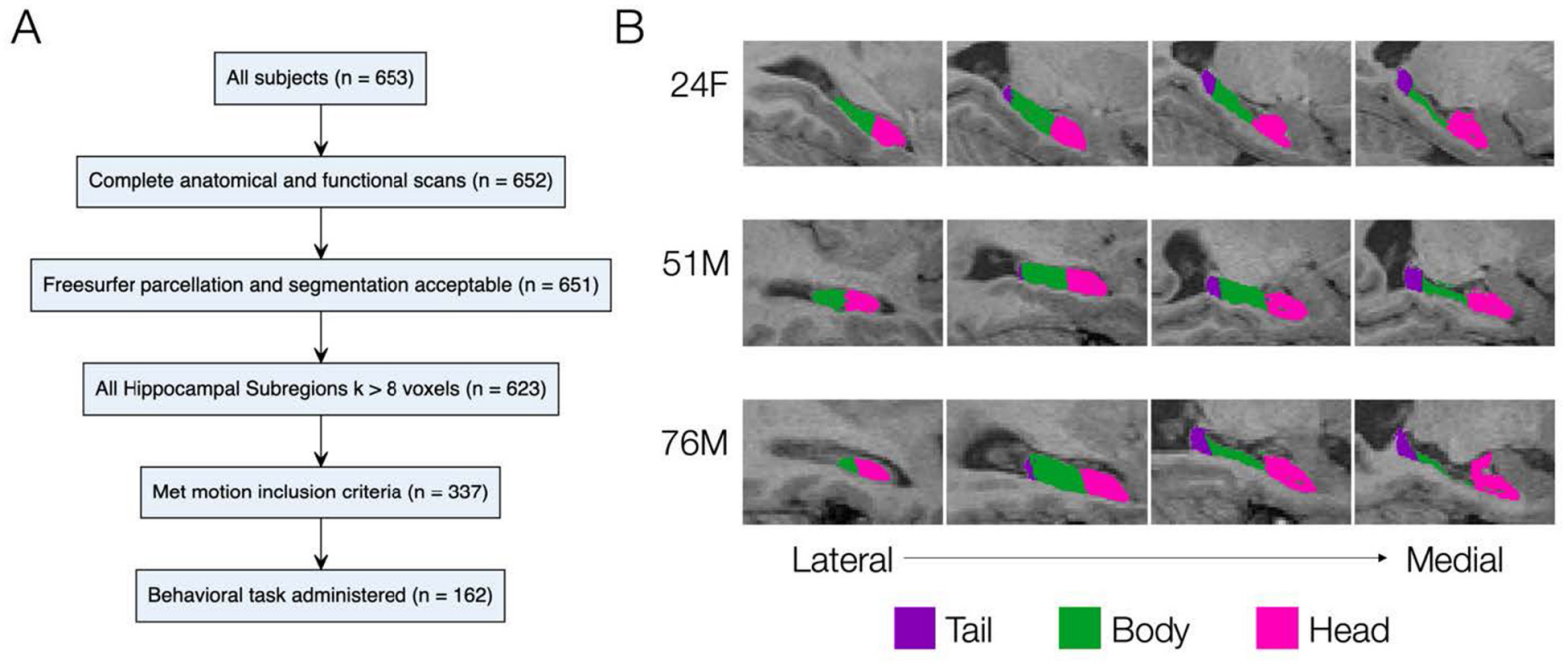
Subject exclusion flowchart and hippocampal subregions of interest. **(A)** Subjects were excluded for missing imaging data, poor quality anatomical segmentation, too few voxels in one or more hippocampal subregions, and excessive motion. Only a subset of the subjects underwent the behavioral memory task and were included in analyses involving links to behavior. Sample sizes at each step represent the number of subjects retained. **(B)** The hippocampal subregions from one randomly selected young adult, middle aged adult, and older adult are depicted on each subject's structural scan. The hippocampus was divided into three segments with the tail (purple) serving as the most posterior region, the body (green) serving as an intermediate region, and the head (pink) serving as the most anterior region.

All data came from the publicly available CamCAN dataset (Shafto et al., 2014). From the larger dataset, we obtained resting state fMRI scans, anatomical MRI scans, and behavioral data from 653 individuals aged 18–88. To ascertain cognitive health, all participants underwent cognitive testing with the Mini-Mental State Exam (MMSE). Those who scored >24/30 points and who did not report any neurological conditions were invited to participate in the fMRI portion of the study. Of the 653 participants we obtained from the database, we excluded subjects for the following reasons (see Figure 1A for subsample size at each step): missing anatomical or functional data, poor segmentation of the hippocampus, too few voxels in one or more hippocampal subregion (minimum voxel threshold set at k = 9 voxels, see supplement for further details), and excessive motion in the functional data. In terms of motion, we completely excluded any subject with any single framewise displacement value greater than or equal to 1 mm or who had fewer than 5 min of data included after scrubbing (details below). These strict motion exclusions were necessary because functional connectivity analyses of rest data are especially sensitive to motion (Power et al., 2012, 2014). The final sample for analyses involving only the neuroimaging data was 337 subjects. Because the emotional memory task that served as the best collected measure of episodic memory abilities was only administered to half of the participants in the original CamCAN study, a subset of the sample used for neuroimaging analyses was carried forward to analyses linking brain indices to behavior. The sample for brain-behavior analyses included 162 subjects. For further details about the distribution of age, sex, education and MMSE scores in these samples, see Table 1.

**Table 1:** Sample characteristics separated by decades-based age groups.

Age group	fMRI data only analyses				Brain-behavior analyses			
	N included	% Female	Mean MMSE	Mean years education	N included	% Female	Mean MMSE	Mean years education
18–29	51	55%	29.2	15.3	26	46%	29.4	15.4
30–39	67	48%	29.2	16.0	31	52%	29.3	15.8
40–49	71	46%	29.2	15.8	33	55%	29.3	15.9
50–59	47	62%	29.4	15.7	25	60%	29.6	16.1
60–69	49	41%	28.8	14.2	20	40%	28.9	14.1
70+	52	38%	28.1	14.2	27	37%	28.2	13.7
All	337	48%	29.0	15.3	162	49%	29.1	15.2

### 2.3 Data acquisition and processing

Participants in the Cam-CAN study completed a series of behavioral tasks across a Stage 1 interview phase. They then completed a Stage 2 phase involving detailed cognitive testing and MRI. Testing occurred over three sessions (Shafto et al., 2014).

#### 2.3.1 MRI data acquisition

Full details of the MRI acquisition protocol have been previously reported (Shafto et al., 2014). From the larger dataset, we used the resting state functional run and the T1-weighted and T2-weighted structural images. During the resting state functional run, participants were asked to keep their eyes closed while 261 EPI volumes were acquired (TR = 1.97 s, TE = 30 ms, 3 mm × 3 mm × 4.44 mm voxel), with a total acquisition time of 8 min and 40 s.

#### 2.3.2 Anatomical data preprocessing and regions of interest selection

We defined anatomical regions of interest in each subject's native space from Freesurfer 7.1.1 (Fischl, 2012). We applied Freesurfer's standard cortical parcellation and subcortical segmentation to the T1-weighted anatomical image. Afterwards, we further segmented the hippocampus by applying Freesurfer's segmentHA_T2 protocol using both the T1-weighted and T2-weighted anatomical images as inputs (Iglesias et al., 2015). Freesurfer's automated hippocampal segmentation was developed from manual segmentations of ultra-high resolution *ex vivo* data and high-resolution *in vivo* data with a protocol developed from several sources (De Nó, 1934; Duvernoy et al., 2013; Green and Mesulam, 1988; Insausti and Amaral, 2012; Rosene and Van Hoesen, 1987; Van Leemput et al., 2009). Prior work has shown good to excellent reliability of these segmentations (Kahhale et al., 2023). From the hippocampal segmentation, we selected the tail, body, and head (separately for the right and left hemispheres) as our posterior, intermediate, and anterior hippocampal regions of interest (ROIs), respectively (Figure 1B). In this segmentation protocol, the boundary between the tail and the body is defined by the point where the fornix fully connects to the hippocampus (Iglesias et al., 2015). The boundary between the body and the head is defined by the caudal boundary of the uncus (Van Leemput et al., 2009). Because some findings have suggested that Freesurfer's overall hippocampal segmentation may perform differently across age groups (Srinivasan et al., 2020), we verified that our findings held when using a multi-atlas segmentation protocol to define the hippocampus (Doshi et al., 2016). The resulting timeseries were highly correlated across hippocampal segmentation methods (within-subject Pearson's correlation M_left_ = 0.934, SD = 0.066; M_right_ = 0.938, SD = 0.072). Although the correlation between Freesurfer- and MUSE-defined hippocampi was lower in older age, β = −0.006, *SE* = 0.001, *t* = −5.31, *p* < 0.001, the correlations were still very high in those older than 60 (M_left_ = 0.910, SD = 0.090; M_right_ = 0.909, SD = 0.108). Further, we found that the temporal signal-to-noise-ratio (tSNR) was numerically higher for Freesurfer-defined hippocampi (see Supplementary materials for details). Thus, we retained the Freesurfer-defined hippocampal segmentations.

We also used the cortical parcellation and subcortical segmentation from Freesurfer to define anatomical regions of interest to serve as targets in the functional connectivity analysis. We used 42 distinct anatomical labels (84 total regions across left and right hemispheres) from the Desikan-Killiany cortical atlas and the subcortical segmentation (excluding the hippocampus since it served as the seed). Although Desikan-Killiany atlas is not as granular as some atlases (e.g., Glasser et al., 2016), it is well-established in the field making our region definitions comparable to many other studies. It also balances coverage of cortical regions while having a manageable number of regions to consider and correct for. For a full list of target regions, see Supplementary Table 1. Compared to a whole brain, voxelwise approach, this approach averages across many voxels to increase the reliability of the signal in each ROI and reduces the number of pairwise connections to correct for when multiple comparison corrections are needed. Using a whole-brain ROI approach also aids in interpreting findings by allowing us to leverage the known structural and functional properties of these regions (Ritchey and Cooper, 2020). After defining ROIs from the structural images, we used the mutual information algorithm from Advanced Normalization Tools (ANTS) version 2.6.3 to register and transform the anatomical images to functional space.

#### 2.3.3 fMRI preprocessing

We first stripped the skull from each subject's resting state functional run using the Brain Extraction Tool from FSL (Jenkinson et al., 2006), then used FSL's MCFLIRT to motion correct the functional data, realigning all volumes to the middle volume. Because functional connectivity measures can be especially sensitive to motion (Murphy et al., 2013; Power et al., 2012), we used the fMRI quality assurance tool (https://github.com/poldrack/fmriqa) to identify subjects who moved excessively as well as individual timepoints with excessive motion. We computed framewise displacement (FD) scores, the temporal derivative of the signal variance over voxels (DVARS), translational and rotational motion, and the temporal derivatives of translational and rotational motion. We excluded all data from any subject who had any single FD value > 1 mm. For the remaining subjects, we identified individual timepoints with movement FD >0.5 mm or DVARS >0.5% and removed those timepoints as well as the timepoint immediately prior and two timepoints immediately after each motion-flagged timepoint. Subjects for whom fewer than 42% of their timepoints were usable (i.e., 5 min of data included) were excluded entirely.

Using FEAT (fMRI Expert Analysis Tool) in FSL, the brain extracted, and motion corrected functional images were subjected to a bandpass temporal filter (0.01-0.08 Hz; Kumar et al., 2024). Functional images were then minimally spatially smoothed using a 2-mm FWHM kernel. We used the 12 translational and rotational motion parameters, plus the mean signals from white matter, CSF, and whole brain and their temporal derivatives as volume-by-volume motion covariates when computing inter-voxel similarity within hippocampal subregions and functional connectivity between hippocampal subregions and the rest of the brain. The bandpass filter was also applied to the nuisance variables prior to computing inter-voxel similarity and functional connectivity.

#### 2.3.4 Inter-voxel similarity

As in prior studies, we computed the correlation across timeseries for each pair of voxels within each hippocampal region (“inter-voxel similarity”; Brunec et al., 2018; Thorp et al., 2022). Higher values on this metric indicate that the voxels within a given hippocampal subregion have more similar signals to one another, with higher similarity proposed to subserve integration across memories and generalization as opposed to specificity and discrimination (Bowman and Zeithamova, 2018; Brunec et al., 2018; Collin et al., 2015; Poppenk et al., 2013; Schlichting et al., 2015). Following pre-processing, we used the fslmeants function from FSL to extract a time course from each voxel within each hippocampal subregion separately from the right and left hemisphere of each subject. We then used custom R code (R Core Team, 2021) to compute the partial correlation between the timeseries of each pair of voxels in each subregion, controlling for volume-by-volume motion estimates. Following a Fisher's Z transformation of the resulting *r*-values, we averaged across the pairwise *z*-values for each subregion, leading to six inter-voxel similarity values for each subject (3 hippocampal subregions × 2 hemispheres).

#### 2.3.5 Functional connectivity

We used the fslmeants function from FSL to extract a mean time course from each of the 84 target regions of interest (42 labels × right and left hemisphere) derived from the Desikan-Killiany atlas (Desikan et al., 2006). We then calculated the functional connectivity scores as a partial correlation between each hippocampal seed region (right and left hippocampal head, body, and tail = 6 seed regions) and each target region (right and left of 42 cortical and subcortical targets = 84 target regions), controlling for volume-by-volume motion estimates. This procedure led to 504 pairwise connections for each subject. We then Fisher's Z transformed the resulting *r*-values and submitted these connectivity values to group-level analyses.

#### 2.3.6 Episodic memory measure

From the cognitive measures obtained, only the emotional memory task specifically tested episodic memory, and we therefore used it as our episodic memory measure. Only half of the participants in the study were administered this task (the other half did an emotion regulation task instead), leading to 162 individuals who were included in imaging analyses and had episodic memory scores available. Full details of the emotional memory task are provided in the Supplementary materials. Briefly, participants first studied objects superimposed on either a negative, neutral, or positive background picture. On each trial of the test phase, participants were tested on perceptual priming, object recognition, and memory for the valence of the image associated with each object. We focused on the object recognition and associative memory components of the test. To test object recognition, participants saw images of objects and were asked whether it was old (presented in the study phase) or new, also indicating their confidence. To test associative memory, participants were asked the valence (positive, neutral, or negative) of the background image presented with the object.

**Figure 2:**
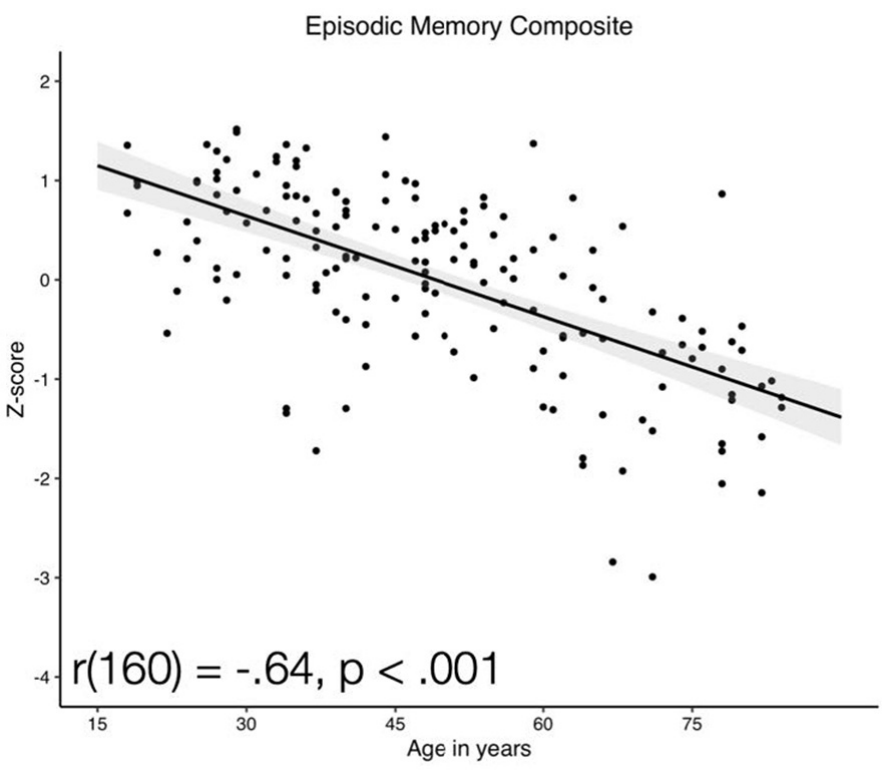
Relationship between age and overall episodic memory performance. The relationship between age and the episodic memory composite derived from the object-emotional background task. The *y*-axis for the composite is the mean *z*-score across the two measures included in the composite (object recognition, and background valence memory). The *r* value represents the zero-order correlation between age and the composite score.

From this task, we sought to develop a single episodic memory outcome measure that was sensitive to age-related memory decline. Although there are known differences in the neural systems supporting single object vs. associative memory, the object recognition and background image valence memory tasks were highly correlated with one another at the individual differences level in this sample, *r*_(160)_ = 0.73, *p* < 0.001. Thus, we reasoned that we would be unlikely to detect subtle differences between their neural correlates in resting state data. We therefore created an episodic memory composite score by calculating *z*-scores separately for the corrected hit rates (proportion hits—proportion false alarms) computed for object recognition and background image valence memory. We then averaged across the two resulting *z*-scores for each participant (Figure 2). The resulting composite measure had a numerically stronger relationship to age [*r*_(160)_ = −0.64, *p* < 0.001] than either of the individual measures [object *r*_(160)_ = −0.57, *p* < 0.001; background *r*_(160)_ = −0.62, *p* < 0.001, Supplementary Figure 3], indicating that it robustly captured age-related memory decline. Although emotion is a factor affecting hippocampal long axis specialization (Bannerman et al., 2003; Fanselow and Dong, 2010), our primary focus was on episodic memory writ large, including memory for both more and less emotional information. Further, memory scores were highly correlated across valence conditions (see Supplementary Figure 4), meaning that we were not able to separate unique effects of emotional above-and-beyond general episodic memory effects. Thus, we averaged across valence conditions in computing scores for both the object and background image valence memory.

### 2.4 Statistical analyses

Group-level statistical analyses were computed in R (R Core Team, 2021) and the full analytic code is available in an OSF repository (https://osf.io/qba8n/). We included participant gender (dummy coded as female = 0, male = 1), subject-level motion (defined as the proportion of excluded timepoints), and overall bilateral hippocampal volume (measured as the proportion of total intracranial volume) as covariates unless otherwise noted. To reduce potential collinearity among predictors, we mean centered continuous variables. We used total hippocampal volume as the covariate to account for general hippocampal decline that was not related to hippocampal subregions differences. We did not include the volumes of hippocampal subregions as covariates because they were moderately to strongly correlated with one another (tail-body r = 0.56, body-head r = 0.62, tail-head r = 0.46), potentially leading to issues of collinearity in the models. As a result, some of the functional differences identified could be related to differential volumetric decline across subregions. Indeed, we would expect differences in function to be the consequence of differences in brain structure at some level (Suárez et al., 2020).

Linear mixed effects models were computed using the nlme package in R (Pinheiro et al., 2025). To determine the best random effects structure for each model, we used Restricted Maximum Likelihood and selected the model with the lowest AIC value. To ensure that models were not affected by multicollinearity issues, we used variance inflation factors (VIF) computed by the car package in R (Fox and Weisberg, 2018). All final models had all VIFs < 10. For *post-hoc* comparisons of specific effects, we corrected for multiple comparisons using the Benjamini-Yekutieli (BY) method, which is a False Discovery Rate correction that accounts for dependencies among multiple tests (Benjamini and Yekutieli, 2001).

#### 2.4.1 Group-level intervoxel similarity models

For testing age effects on intervoxel similarity across the hippocampal long axis, the final linear mixed effects model included age, hippocampal subregion (tail, body, head), and hemisphere (right, left) as predictors of interest, along with their interactions. The hippocampal head was used as the reference group in the model both because we predicted that it would remain more stable with older age compared to other regions and because our initial assessments of the raw data aligned with this prediction. Gender, hippocampal volume, and motion were included as covariates. The final model included a random intercept plus random slopes for hemisphere and hippocampal subregion. We focused on overall differences across hippocampal subregions at three different levels of age (30, 50, and 70 years), significant age trends within each hippocampal subregion and differences in the age slopes between hippocampal subregions. Next, we used multiple linear regression to determine the relationship between intervoxel similarity and episodic memory performance. We computed a model with age, IVS in each hippocampal subregion separated by hemisphere, interactions between age and IVS in each subregion, and standard covariates.

#### 2.4.2 Group-level functional connectivity models

To test for age-related differences in patterns of functional connectivity along the hippocampal longitudinal axis, we computed functional connectivity between each hippocampal subregion and 42 cortical and subcortical regions in each hemisphere (84 total targets across hemispheres) defined from the Desikan-Killiany Atlas (Desikan et al., 2006). We took two main approaches to the whole-brain functional connectivity data. First, we sought to test the similarity of whole-brain connectivity profiles for each pair of hippocampal subregions. This approach was similar to representational similarity analyses that are typically used to determine the similarity between patterns of functional activation (Kriegeskorte et al., 2008), substituting patterns of connectivity strength across target regions. Our second approach focused on differences in the strength of individual connections with target ROIs across hippocampal subregions.

##### 2.4.2.1 Similarity of connectivity profiles

In each individual subject, we computed the correlation between the unthresholded pattern of connections for each pair of ipsilateral hippocampal segments (e.g., left tail connectivity map similarity to left body connectivity map). This process resulted in six Fisher's Z transformed correlation values for each subject (tail-body, body-head, tail-head for left and right hippocampus). To test for differences in the degree of connectivity similarity between subregions of the hippocampus, we submitted these connectivity similarity values to a linear mixed effects model that included age, the region comparison (tail-body, body-head, tail-head), hemisphere, and all interaction effects as predictors of interest alongside standard covariates. The final model included a random intercept, plus random slopes for the region comparison and hemisphere. We focused on differences in similarity across pairs of subregions at three different levels of age (30, 50, and 70 years), age trends for each subregion comparison, and differences in the age slopes across subregion comparisons. We then used multiple linear regression to determine the relationship between the similarity of connectivity profiles and episodic memory performance. We started with a model that included age, connectivity similarity values for each pair of hippocampal subregions separately for each hemisphere, interactions between age and connectivity similarity for each subregion comparison for each hemisphere, and standard covariates.

##### 2.4.2.2 Individual functional connections

We tested for differences in the strength of functional connectivity across the hippocampal long axis and age moderation of connectivity strength for each target region. To do so, we conducted separate linear mixed effects models for each target ROI (42 in total). For these models, we averaged across hemispheres to reduce the complexity and improve the stability of estimates. Specifically, considering hemisphere effects would dramatically increase the number of predictors because there would be an effect of hippocampal subregion hemisphere and an effect of target region hemisphere, plus age, hippocampal subregion, and corresponding interaction effects, leading to 15 predictors of interest plus standard covariates in the full model, reducing the likelihood of model convergence and hindering interpretability. Averaging across hemispheres resulted in models with only 3 predictors of interest, sacrificing some specificity to the findings but aiding model stability. Thus, we included age, hippocampal subregion (head as the reference as above), and their interaction as predictors of interest alongside the standard covariates. We included only a random intercept because of the reduced within-subjects data. We were particularly interested in connections where the age slopes differed across hippocampal subregions and computed pairwise comparisons of these slopes for each model, performing a Benjamini-Yekutieli (BY) correction across all comparisons across all models.

##### 2.4.2.3 Predicting episodic memory with a combination of functional connections

Lastly, we conducted an exploratory analysis seeking to identify a set of hippocampal connections that could predict episodic memory performance. We computed three types of models to predict episodic memory performance that differed in the predictors available in addition to standard covariates: an “Age only” model that included only age in addition to standard covariates, a “Connectivity only” model that entered all hippocampal subregion—target ROI connections as potential predictors but not age, and a “Full” model that combined the age and connectivity models and added all age × connection interaction effects as potential predictors. Because of the large number of potential predictors in the Connectivity only and Full models, we used a regularized regression approach to select predictors for the model (using the glmnet package in R; Friedman et al., 2010; Tay et al., 2023). We used LASSO regression, which allows coefficients to be set to zero to allow for selection of relevant predictors (Tibshirani, 1996). We also used a cross-validation approach, splitting the data set into 10 cross-validation folds that were stratified by age such that each fold included approximately the same number of participants from each decades-based age group. For each fold, the model was trained on 9 of 10 of the folds and tested on the left-out fold. For each model type and each fold, we obtained predicted episodic memory scores for the participants in the test set based on the model developed from the training set. We then compared the model predicted episodic memory scores to the observed scores, generating an *R*^2^ value reflecting how well the model predicted the participants' real scores. We repeated this 10-fold cross-validation process 10 times to provide sufficient data points to compare the three types of models (total observations per model = 100). We then used the estimated marginal means from a linear mixed effects model to compare each model's *R*^2^ values to 0 (representing the predictive power of the mean episodic memory score from the training set), and to compare across model types. The linear mixed effects model included a fixed effect of model type (Full, Connectivity only, Age only) and random intercepts for repetition and fold, with fold nested within repetition.

## 3 Results

### 3.1 Age-related differences in within hippocampal signals

**Figure 3:**
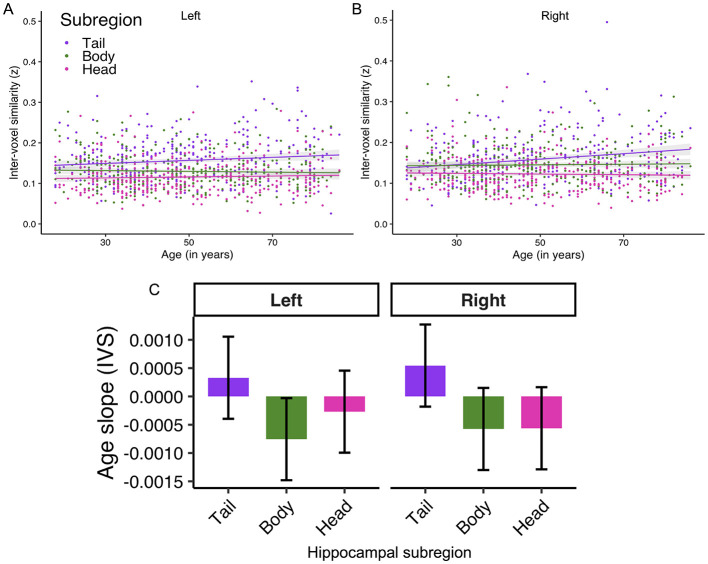
Age-related differences in the correlation of within-hippocampal signals. The relationship between continuous age (in years) and inter-voxel similarity in each hippocampal subregion in the **(A)** left and **(B)** right hemispheres. Trendlines represent the relationship between age and intervoxel similarity prior to consideration of covariates. **(C)** Estimated age slopes from the linear mixed effects model in each hippocampal subregion separated by hemisphere. In **(A–C)**, the shaded regions and error bars represent 95% confidence intervals.

Our first approach to understanding the impact of age on hippocampal functional specialization was to compare signals from within each of the three hippocampal regions using intervoxel similarity. For descriptive purposes, Figures 3A, B depict the relationship between age and intervoxel similarity separated by hippocampal subregion and hemisphere prior to considering any covariates. There was a notable outlier for tail intervoxel similarity in the right hemisphere, but the pattern of results did not differ based on the inclusion or exclusion of that subject, and they were retained. We then computed a linear mixed effects model that included age, hippocampal subregion, hemisphere, and all interaction effects as predictors of interest. From Figure 3, it seemed that there was a long axis gradient in signal similarity, but with the most posterior region having higher within-region signal similarity than more anterior regions. We confirmed this pattern by computing pairwise comparisons of the estimated marginal means between subregions at three age levels: 30, 50, and 70 years, separately for each hemisphere (no cross-hemisphere comparisons computed). A similar tail > body > head numerical pattern emerged for each age and in both hemispheres. All pairwise comparisons between subregions reached significance in the left hemisphere (all corrected *p*'s < 0.03), with the exception of the head vs. body comparison at the youngest age (corrected *p* = 0.75). Results were more mixed in the right hemisphere: at the youngest age, no pairwise comparisons were significant (all *p*'s > 0.43); at the middle age, only the difference between the body and the tail reached significance (corrected *p* = 0.006; other *p*'s > 0.06); and in the oldest age, the tail differed significantly from both the body and head (corrected *p*'s < 0.03), but the head and body did not differ significantly from one another (corrected *p* = 0.26). Thus, we found a gradient in within hippocampal signals in the opposite direction from the Brunec et al. (2018) study and closer to the pattern from Thorp et al. (2022): signals within the most posterior hippocampal (tail) subregion were most similar to one another, followed by the intermediate (body) region, and signals in the most anterior (head) region were the least similar to one another. This pattern was most prominent in the left hemisphere and at older ages.

Next, we tested for age-related change in the similarity of signals within each subregion in each hemisphere by comparing the model estimated slopes to 0 (no age-related differences). Figure 3C depicts the model estimated age slopes. Only the age effect in the left hippocampal body reached significance (β = −0.001, SE = 0.004, corrected *p* = 0.04; all other corrected *p*'s > 0.11), indicating significant age-related decreases in the similarity of signals within this region. While we did not include subregion-specific volumes in the linear mixed effects model due to concerns of multicollinearity, we computed a partial correlation between age and IVS in the left hippocampal body that included body-specific volume in place of the overall hippocampal volume alongside other standard covariates. Age-related differences became marginal but were in the same direction (partial *r* = −0.103, *p* = 0.059), suggesting some explanatory power of volumetric differences that still leave some evidence of age-related differences in the similarity of signals within the left hippocampal body.

We then tested whether there were differences in the age-related trajectories across hippocampal subregions by computing pairwise differences between the estimated age slopes across subregions within each hemisphere. In the left hemisphere, the difference between the tail and the body reached significance (corrected *p* = 0.04; other *p*'s > 0.38), with signals within the body becoming less similar to one another with older age and signals within the tail becoming numerically more similar to one another with older age. In the right hemisphere, signals within the tail increased more than both the body and head with older age significantly (both corrected *p*'s < 0.02). Age slopes did not differ significantly between the body and head (corrected *p* > 0.99). We tested the extent to which subregion-specific volumetric differences accounted for the positive direction of the effect in the tail compared to the body and head. Partial correlations between age and intervoxel similarity in each subregion remained in the same direction in each region even after accounting for subregion-specific volumes. Thus, we found evidence that the hippocampal tail was on a different age-related trajectory than more anterior hippocampal regions, with signals becoming numerically more similar to one another while signals within more anterior regions became more distinct in older age.

Next, we sought to relate differences in within-hippocampal signals to differences in episodic memory performance. To do so, we computed a multiple linear regression that included age, intervoxel similarity from each hippocampal subregion in each hemisphere, and interactions between age and intervoxel similarity in each subregion as predictors of composite episodic memory scores. Full results from the model are presented in Supplementary Table 2. While the overall model was quite successful in accounting for differences in episodic memory, *F*_(16, 145)_ = 8.51, *p* < 0.001, adjusted *R*^2^ = 0.48, none of the individual IVS predictors were significant, and the strong negative relationship between age and episodic memory remained significant. Further, the IVS and age model was not a significant improvement over an age only model, *F*_(4, 157)_ = 30.09, *p* < 0.001, adjusted *R*^2^ = 0.42, model difference *F*_(12, 145)_ = 1.18, *p* = 0.30. However, when we averaged across hemispheres to reduce the number of predictors in the model, we found significant relationships between episodic memory scores and IVS in the body and head (see Supplementary Table 3 for full model results). Higher similarity of signals within the hippocampal body was associated with poorer episodic memory scores, although this relationship was no longer significant when we removed one outlier, β = 1.279, *t* = −1.57, *p* = 0.119. Higher similarity of signals within the hippocampal head was associated with better episodic memory, and this relationship remained when the outlier subject was removed, β = 1.672, *t* = 2.57, *p* = 0.011. Once again, however, the relationship between age and episodic memory remained, and the model using bilateral hippocampal subregions was not a significant improvement over an age only model, *F*_(10, 150)_ = 13.43, *p* < 0.001, adjusted *R*^2^ = 0.47, model difference *F*_(6, 151)_ = 4.77, *p* = 0.14. Thus, there was some evidence that similarity of signals within hippocampal subregions predicted episodic memory, but within-hippocampal signals did little to account for age-related episodic memory decline.

### 3.2 Age-related differences in hippocampal-whole brain functional connectivity

#### 3.2.1 Similarity in connectivity profiles

**Figure 4:**
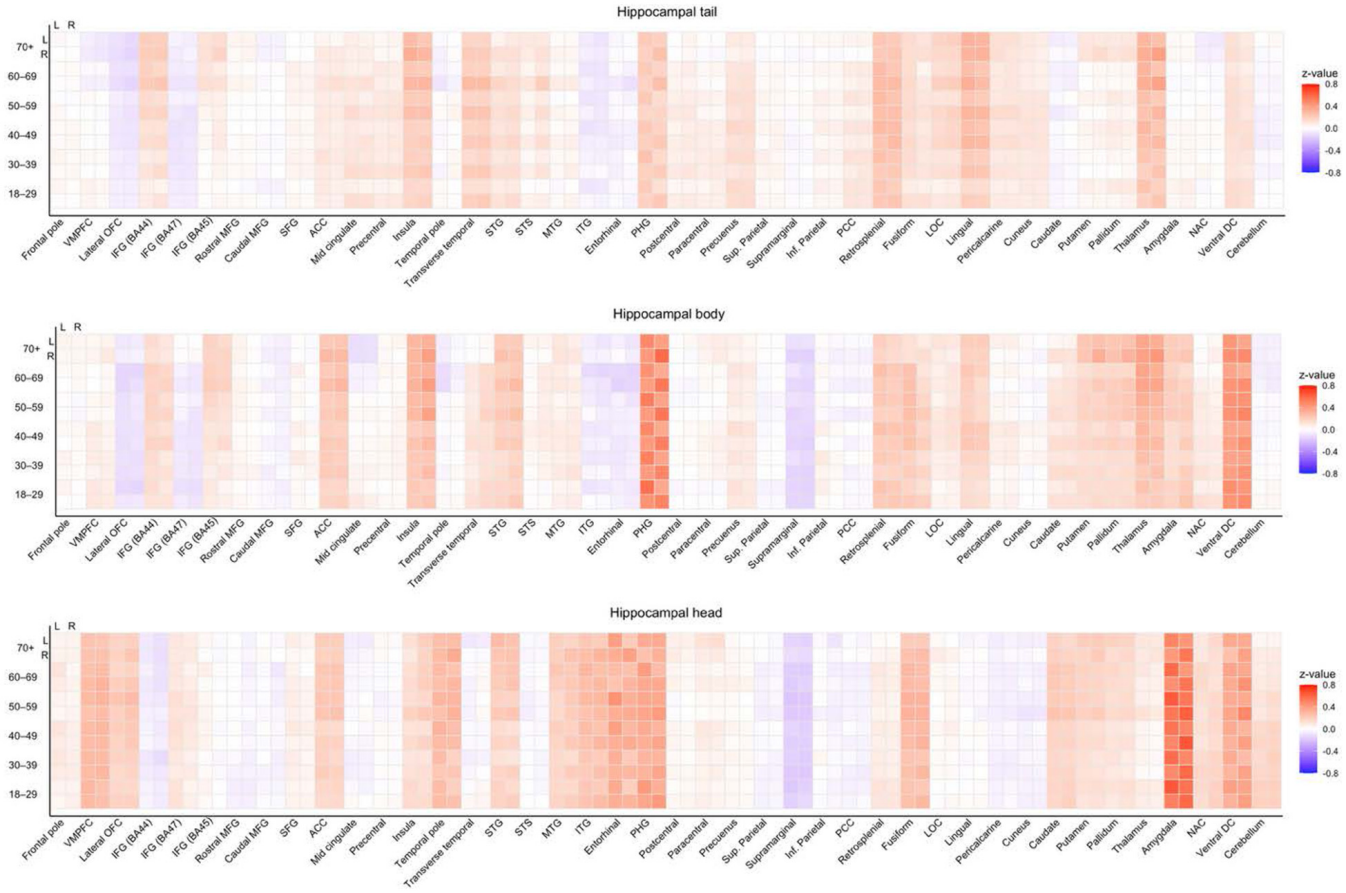
Functional connectivity for each hippocampal subregion and target region across the adult lifespan. Functional connectivity values presented separately for the hippocampal tail, hippocampal body, and hippocampal head. Functional connectivity was computed as the Fisher-z transformed partial correlation between timecourses in each hippocampal seed region and 84 cortical and subcortical target regions across both hemispheres. Darker red colors represent stronger positive z-values, darker blue colors represent stronger negative z-values, and white represents z-values near zero. Participants were separated into categorical age groups to visualize age effects. Left (L) and right (R) hippocampal seeds are presented in adjacent rows, and left and right target regions are presented in adjacent columns. Full names and atlas labels for abbreviated target regions are presented in Supplementary Table 1.

Looking beyond within-hippocampus signals, we computed functional connectivity between each hippocampal subregion and ROIs across the rest of the brain. For descriptive purposes, Figure 4 depicts functional connectivity values for all target regions separated by hippocampal subregion and approximately decade-based age groups (18–29, 30–39, 40–49, etc.). Our first approach to quantifying patterns of hippocampal connectivity was to determine the degree of similarity in the whole-brain connectivity profiles of each pair of hippocampal subregions and whether similarity in connectivity profiles differed across the adult lifespan. To test for differences in the degree of connectivity similarity between subregions of the hippocampus, we submitted these connectivity similarity values to a linear mixed effects model that included age, the region comparison (tail-head, tail-body, body-head), hemisphere, and all interaction effects as predictors of interest alongside standard covariates.

**Figure 5:**
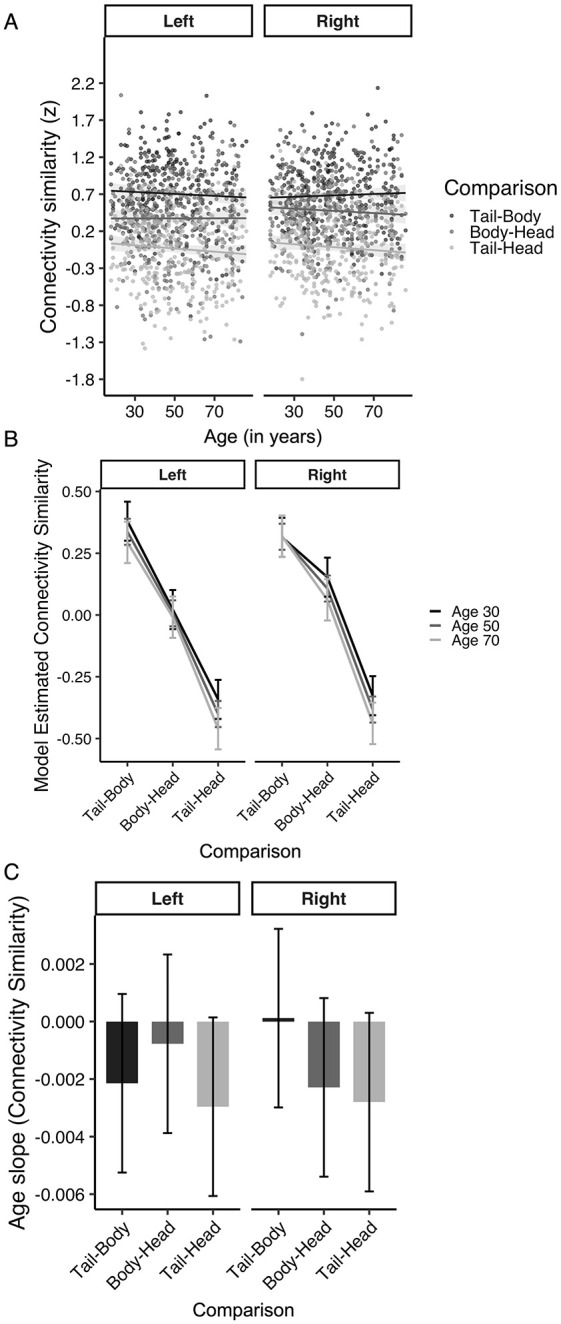
Similarity in connectivity profiles between hippocampal subregions. **(A)** The relationship between continuous age (in years) and similarity in unthresholded functional connectivity profiles between the tail and body, body and head and tail and head prior to consideration of covariates. Similarity values are the Fisher's Z transformation of the Pearson's correlation between the connectivity values for all target regions across both hemispheres associated with each pair of hippocampal subregions. **(B)** Estimated marginal means of connectivity similarity for each subregion comparison depicted separately for each hemisphere and at three levels of age: 30, 50, and 70 years. **(C)** Model estimated age slopes for connectivity similarity between hippocampal subregions. In **(A–C)**, error bars/shaded regions represent 95% confidence intervals.

For descriptive purposes, Figure 5A depicts the raw connectivity similarity values across age and separated by hemisphere prior to consideration of covariates. Figure 5B depicts the model estimated marginal mean connectivity similarity for each subregion comparison at ages 30, 50, and 70 separated by hemisphere. Across ages and hemispheres, there was a clear gradient in similarity. Pairwise comparisons of the marginal means confirmed that the tail and body had more similar connectivity profiles than the body and head across ages and across hemispheres (all corrected *p*'s < 0.003). The body and head connectivity profiles were significantly more similar than the tail and head connectivity profiles (all corrected *p*'s < 0.001). Lastly, the tail-body vs. tail-head comparison showed the largest difference, with the tail and body connectivity profiles more similar to one another than the tail and head connectivity profiles (all corrected *p*'s < 0.001). While this overall pattern held across hemispheres, there was a significant interaction effect, β = 0.12, *t* = 3.41, *p* = 0.0007. Higher connectivity similarity in the right compared to left hemisphere emerged for the body-head comparison at age 30 and 50 (corrected *p*'s < 0.002), with a non-significant effect in the same direction at age 70 (corrected p = 0.11). Differences across hemispheres did not emerge for the other two comparisons at any age tested (all corrected *p*'s > 0.11). Thus, across age groups and across hemispheres, the connectivity profiles of the extreme posterior and extreme anterior subregions were the most distinct from one another, whereas the intermediate region (body) showed its highest connectivity similarity with the most posterior subregion but still substantial similarity with the most anterior subregion.

Next, we tested the age slopes for each comparison relative to 0 (no age-related differences). Figure 5C depicts the model estimated age slopes separately for each comparison in each hemisphere. In each hemisphere, there was a marginal effect of age for the tail-head comparison (both corrected *p*'s < 0.08), with the tail and head connectivity profiles tending to become marginally less similar to one another in older age. No other age effects approached significance (all other corrected *p*'s > 0.14), and pairwise comparisons between age slopes did not approach significance for any comparison (all corrected *p*'s > 0.57). Thus, there was a clear gradient in the similarity of connectivity profiles along the hippocampal long axis with relatively limited evidence of age-related differences.

We next tested the extent to which the similarity of connectivity profiles between pairs of hippocampal subregions were related to age differences in episodic memory performance. Full results from the model are presented in Supplementary Table 4. There was a marginal positive effect of similarity between the tail and body connectivity profiles, β = 0.26, *t* = 1.85, *p* = 0.066, such that more similar connectivity profiles were associated with marginally better episodic memory performance. No other connectivity similarity effect approached significance (all other *p*'s > 0.14), and the strong negative age effect remained significant, β = −0.031, *t* = −8.78, *p* < 0.001. While the model that included hippocampal connectivity similarity explained significant variance in episodic memory, *F*_(16, 145)_ = 8.90, *p* < 0.001, adjusted *R*^2^ = 0.50, it did not significantly outperform a model with age and standard covariates only, *F*_(4, 157)_ = 30.09, *p* < 0.001, adjusted *R*^2^ = 0.43, model difference *F*_(12, 145)_ = 1.47, *p* = 0.14. We next considered whether including separate left and right hemisphere predictors may have masked a true effect due to correlations between these predictors, running a model that collapsed across hemispheres for the connectivity similarity (Supplementary Table 5). Still, no connectivity similarity predictor reached significance (all *p*'s > 0.25), age remained a robust predictor of episodic memory decline, β = −0.033, *t* = −9.30, *p* < 0.001, and the model did not outperform the age and standard covariates model, *F*_(10, 151)_ = 13.4, *p* < 0.001, *R*^2^ = 0.44, model difference *F*_(6, 151)_ = 1.72, *p* = 0.12. Thus, there was a hippocampal long axis gradient in patterns of whole-brain resting-state functional connectivity, but evidence for age differences in connectivity profiles was weak, and they were not strongly related to individual differences in episodic memory performance.

#### 3.2.2 Age differences in the strength of individual hippocampal subregion connections

**Figure 6:**
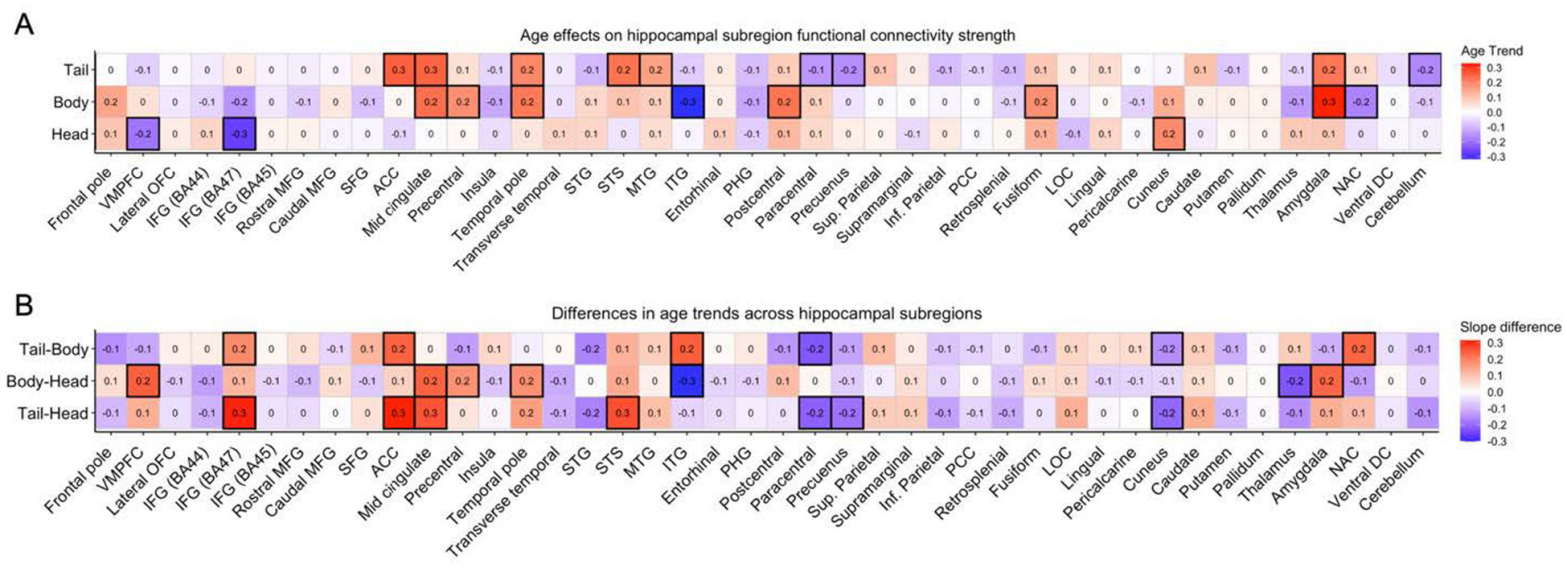
Age differences in the strength of individual hippocampal subregion connections. **(A)** Model estimated age slopes for each target-hippocampal subregion connection. **(B)** Pairwise differences in model estimated age slopes for each target-hippocampal subregion connection. Age trend estimates and differences in age trends were multiplied by 100 for ease of visualization. Darker red colors represent stronger positive age trends and slope differences, darker blue colors represent stronger negative age trends and slope differences, and white represents values near zero. Effects with a black border were significant after a correction for multiple comparisons.

Next, we sought to identify brain regions showing age moderation in the strength of connections across hippocampal subregions. We computed linear mixed effects models for each of the 42 target ROIs, with Age, Hippocampal subregion (reference = hippocampal head), and their interaction as fixed effect predictors. Figure 6A depicts the model estimated age slopes from each hippocampal subregion and each target ROI. The number of connections showing significant age-related differences in strength was smallest for the hippocampal head (*n* = 3), whereas the tail (*n* = 9) and body (*n* = 8) showed a comparable number of connections showing age-related strength differences. Significant increases in connectivity strength with age were identified between the hippocampal tail and anterior cingulate, mid cingulate, temporal pole, STS, MTG, and amygdala; between the hippocampal body and midcingulate, precentral gyrus, temporal pole, postcentral gyrus, fusiform gyrus, and amygdala; and between the hippocampal head and the cuneus. Fewer significant age-related decreases in connectivity strength were identified (*n* = 13 positive effects, *n* = 5 negative effects). Significant decreases in connectivity strength were identified between the hippocampal tail and paracentral cortex, precuneus, and cerebellum; between the hippocampal body and the ITG and NAC; and between the hippocampal head and VMPFC and IFG (BA 47).

We also tested the extent to which age trends in the strength of connections differed across hippocampal subregions. Figure 6B depicts differences in model estimated age slopes for all pairwise comparisons between hippocampal subregions for each target ROI. Positive effects represent comparisons between hippocampal subregions where differences in connectivity strength increased with older age. Negative effects represent comparisons between hippocampal subregions where differences in connectivity strength were less pronounced with older age. Significant differences were relatively equally distributed across the three hippocampal subregion comparisons (*n* = 6–7 per comparison). Multiple frontal regions showed a pattern of increasing differences in connectivity with older age, with the VMPFC, IFG (BA 47), ACC, mid cingulate, and precentral gyrus showing at least one significant positive difference in slopes. No frontal region showed a significant negative difference in age slopes. Other regions showing increased differences in strength of connectivity with increasing age included the ITG and NAC (between tail and body), temporal pole and amygdala (between body and head), and the STS (between tail and head). There were fewer target ROIs showing decreased differences in connectivity strength between hippocampal subregions with age, but they included the paracentral cortex and cuneus (between both the tail and body and the tail and head), the ITG and thalamus (between body and head), and the precuneus (between the tail and head). Taken together, these results indicate that the predominant effect, especially in frontal regions, was increased functional differences between hippocampal subregions with increasing age in terms of connectivity strength. A more limited set of relatively posterior regions showed decreased functional specialization of connectivity strength with older age.

Lastly, we tested the extent to which hippocampal connectivity could explain age differences in episodic memory performance. We first tested whether any individual connections explained the relationship between age and episodic memory scores, computing multiple regressions including age, the strength of an individual hippocampal subregion-target ROI connection, and standard covariates as predictors of episodic memory scores. Unsurprisingly given the complexity of episodic memory and robustness of its age-related decline, none of these models was a significant improvement over an age and standard covariates only model (all corrected *p*'s > 0.96).

**Figure 7:**
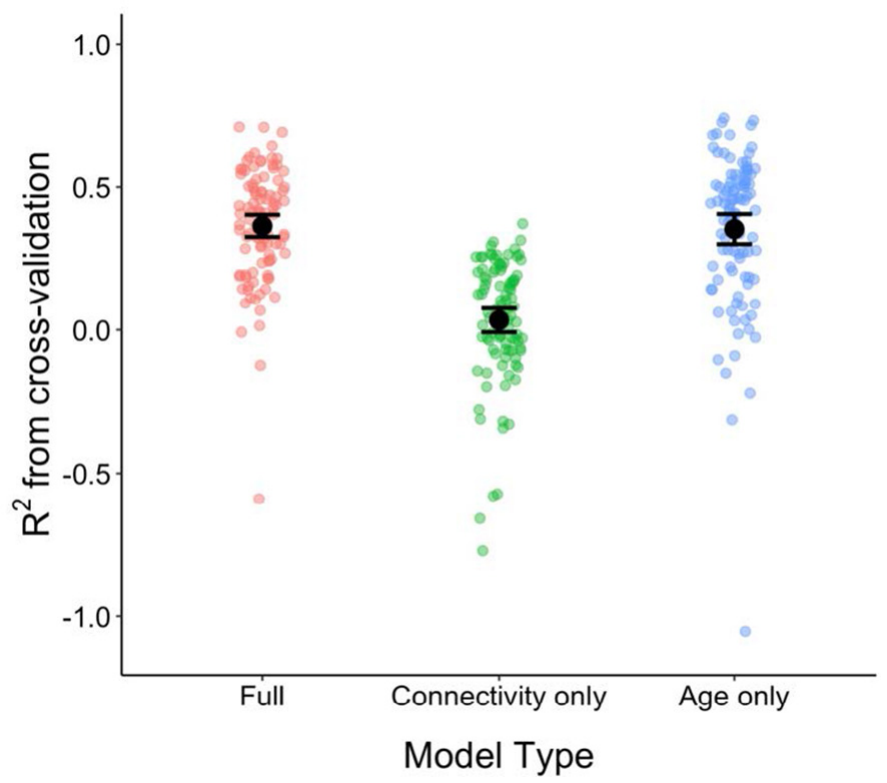
Relationships between episodic memory performance and connectivity strength. *R*^2^ values from cross-validation of regularized regression models predicting episodic memory scores. The full model made age, connectivity between each hippocampal subregion and all target ROIs, and age interactions with hippocampal subregion connectivity available to enter into the predictive model. The connectivity model made only the hippocampal subregion connectivity available without age or age interactions. The age only model made only age available as a predictor without connectivity or age × connectivity interactions. Colored dots represent *R*^2^ values from individual cross-validation folds for each model, black dots represent the mean across folds, and error bars represent the 95% confidence interval across cross-validation folds.

Yet the possibility remained that a *combination* of connections could explain age differences in episodic memory performance. To test that possibility, we used a regularized regression approach to select predictors of episodic memory. We ran three versions of the model that differed in the predictors that were available to be entered in the model: one that included only age (“Age only”), one that included the strength of connectivity for each hippocampal subregion—target ROI pair (“Connectivity only”), and one that included the predictors from the previous models together plus all age × connectivity interaction effects (“Full”). Standard covariates were always included in each model. We used cross-validation to test for generalizability of the model from a training set of subjects to a new testing set of subjects. Figure 7 depicts the *R*^2^ values from model estimated episodic memory scores predicting the observed episodic memory scores in the test portion of the cross-validation sets. Supplementary Figure 5 depicts the mean coefficients for all predictors across cross-validation folds and repetitions. The mean *R*^2^ values for the Full and Age only models indicated substantial predictive power (Full M = 0.36, SD = 0.20; Age only M = 0.35, SD = 0.27), differing significantly from 0 (with 0 representing the predictive power of the mean episodic memory score from training; both *t*'s > 15, corrected *p*'s < 0.0001). The Connectivity only model had relatively poor predictive power (M = 0.03, SD = 0.21) that did not differ significantly from 0, *t*_(9)_ = 1.47, corrected *p* = 0.32. We next tested whether the predictive power of these models differed significantly from one another, computing a linear mixed effects model on the *R*^2^ values, with model type (“Full,” “Age only,” “Connectivity only”) as the main predictor of interest. Pairwise comparisons of the marginal means across model types confirmed that both the Full and Age only models outperformed the Connectivity only model (both *t*'s > 15.8, corrected *p*'s < 0.0001), but that the Full model did not significantly outperform the Age only model, *t*_(198)_ = 0.56, *p* > 0.999. Thus, despite age-related differences in the strength of functional connectivity along the hippocampal long axis for a number of target regions, there was not a strong relationship between these resting state connections and episodic memory performance that could explain age-related episodic memory decline.

## 4 Discussion

We aimed to test the degree of functional differentiation along the hippocampal long axis across the adult lifespan using within-hippocampal signals and functional connectivity with the rest of the brain. We found that within-hippocampal signals in the extreme posterior subregion were on a more positive age trajectory than the intermediate and extreme anterior subregions. While whole-brain connectivity profiles showed little evidence of age-related differences, there were numerous individual connections whose strength differed with older age and target regions whose age trajectory differed across hippocampal subregions, especially in frontal cortex. The most common pattern was increasing differences in connectivity strength with older age. Yet hippocampal functional specialization during rest did not show a clear relationship to episodic memory performance, suggesting that patterns of offline hippocampal subregion function may not reflect an individual's capacity to engage mnemonic processes during a memory task.

One theory of hippocampal function is that there is a posterior to anterior gradient in which the posterior hippocampal representations are more detailed than anterior hippocampal representations (Poppenk et al., 2013). Compelling evidence for this theory came from a study examining the correlation of signals within the hippocampus, showing less correlated signals in the posterior vs. anterior hippocampus (Brunec et al., 2018). The authors concluded that the low correlation among posterior hippocampal signals made the posterior hippocampus well suited to representing information with high resolution to support memory specificity. However, a more recent study tested multiple schemes for subdividing the hippocampus and compared them across two large datasets (Thorp et al., 2022). While there were differences based on the parcellation scheme, results generally pointed to higher similarity among signals in the posterior hippocampus once the hippocampus was subdivided into more than two subregions. Our overall findings are most in line with the latter study: signals within the hippocampal tail were more correlated with one another than signals in the body and the head. One potential driver of differences across studies is that the Brunec et al. (2018) study used both resting state data and data from a navigation task, and the increase in intervoxel similarity from posterior to anterior was more prominent in the navigation task. Our study and the Thorp et al. (2022) study used only resting state data. Thus, it may be that within-hippocampal signals are noisier during rest and show more variable patterns across the hippocampal long axis, but that these signaling patterns emerge when the participant is engaged in a task supported by the hippocampus.

Despite an overall pattern of within-hippocampal signals that differed from predictions, we found that within-hippocampal signaling in the posterior hippocampus had a different trajectory across the adult lifespan compared to intermediate and anterior regions of the hippocampus, which is consistent with what we would predict based on known age-related declines in various measures of memory specificity (Hashtroudi et al., 1989; Naveh-Benjamin et al., 2003; Yassa et al., 2011). Signals within the posterior hippocampus became numerically more similar to one another with increasing age, the opposite of the trend for the intermediate and anterior hippocampal subregions. This finding is consistent with a prior study showing age-related increases in the strength of functional connectivity within posterior medial temporal lobes (Salami et al., 2016). Increasing similarity of signals in the posterior hippocampus is also in line with neurocognitive aging theories suggesting dedifferentiation of neural signals in older age (Goh, 2011; Koen and Rugg, 2019; Park et al., 2012; Pauley et al., 2024): that neural responses become less distinctive in older age. Here we show that this effect may be specific to the posterior vs. intermediate and anterior regions of the hippocampus.

Yet evidence for a functional specialization along the hippocampal long axis has also come from differences in patterns of structural and functional connectivity with the rest of the brain. Prior work has shown that the posterior hippocampus has structural and/or functional connections to regions including retrosplenial cortex, the posterior cingulate, precuneus, angular gyrus, visual cortices, and lateral prefrontal cortex (Frank et al., 2019; Poppenk et al., 2013; Poppenk and Moscovitch, 2011; Ranganath and Ritchey, 2012). In contrast, the anterior hippocampus has shown stronger connectivity with regions including the ventromedial prefrontal cortex, amygdala, and temporal pole (Blessing et al., 2016; Bowman and Zeithamova, 2018; Duvernoy et al., 2013; Frank et al., 2019; Kier et al., 2004; Robinson et al., 2016; Wang et al., 2016; Zeithamova et al., 2012). Qualitatively, these patterns emerged within the current dataset (see Figure 4), but we focused our analyses on the overall similarity of connectivity profiles across three subregions of the hippocampus rather than the individual connections that differed between the posterior and anterior hippocampus. Results showed a clear gradient in the similarity of connectivity profiles, with the greatest similarity between the posterior and intermediate subregions, and the least similarity between the extreme posterior and anterior subregions. While the intermediate subregion is combined with the most posterior region in a common parcellation scheme (Poppenk et al., 2013), it was nonetheless substantially more similar to the most anterior region than the extreme posterior and anterior subregions were to one another. This finding suggests that the connectivity profile of the intermediate subregion is somewhat of a mix of “posterior-like” and “anterior-like” connectivity, albeit biased more toward the “posterior-like” profile. Further, we did not find significant age moderation of the similarity between connectivity profiles. Thus, we found strong evidence for a long axis gradient in whole-brain hippocampal functional connectivity profiles, and the similarity of these profiles remained relatively stable across the lifespan.

We also examined age-related trajectories in the strength of individual connections with hippocampal subregions and differences between hippocampal subregions in these trajectories. We also found that older age moderated the pattern of connectivity across hippocampal subregions for many target regions. Age-related differences in connectivity strength were more prevalent for the more posterior hippocampal subregions (both tail and body) relative to the head, with the head showing fewer significant age effects. These findings are in line with the hypothesis that relatively posterior hippocampal regions are the most impacted in older age (Bussy et al., 2021; Frisoni et al., 2008; Xiao et al., 2023). Yet they are novel in showing that the net result for many target regions was more pronounced differentiation of connectivity strength across hippocampal subregions with increasing age. This age-related increase in differentiation of connectivity strength was particularly prominent across frontal cortex, with VMPFC, IFG (BA 47), ACC, and mid cingulate showing this pattern for at least one comparison between subregions and showing no significant negative age effects between subregions. These findings are in line with work showing that frontal regions often show the largest age-related differences in function (Cabeza and Dennis, 2012; Davis et al., 2008; West, 1996), which has sometimes been linked to compensation (Reuter-Lorenz and Cappell, 2008) and sometimes linked to dysfunction (Daselaar et al., 2015). In contrast, target regions that showed reduced differences in connectivity strength across hippocampal subregions (i.e., negative differences in age slopes) tended to be in more posterior regions of the brain and included the precuneus and the cuneus. These findings are in line with neurocognitive theories proposing that aging reduces the specificity of sensory processing and level of sensory details in memories (Bowman et al., 2019; Davis et al., 2008; Dennis et al., 2014; Koen and Rugg, 2019; Park et al., 2004). Taken together, investigation of individual hippocampal connections pointed toward relative stability of connectivity for the anterior hippocampus, and a tendency toward increasing differentiation of connectivity strength across hippocampal subregions with increasing age, with a smaller number of targets showing decreasing differentiation.

Looking across analytical approaches, there was an apparent paradox: within-posterior hippocampus signals showed signs of age-related dedifferentiation even as many connectivity targets showed increasing differentiation across hippocampal subregions with older age. Signals within the most posterior subregion became relatively more similar to one another compared to the intermediate and anterior subregions, which is consistent with the posterior hippocampus losing some of the heterogeneity in its signal. However, it does not seem that within-hippocampal signals were becoming more similar *across* subregions because, if that were the case, we would expect whole-brain connectivity profiles to converge across hippocampal subregions alongside the convergence of within-hippocampal signals. Instead, we found that the overall connectivity profiles of hippocampal subregions did not show different age-related trajectories, and that many individual target regions, especially in frontal cortex, showed increases in preferential connectivity across hippocampal subregions in older age. This pattern could emerge if the signal changes within the posterior hippocampus converged toward the most distinctly “posterior” signals, enhancing the most distinct functional connections with the rest of the brain while also making the signals with the subregion more consistent with one another. However, future research will be necessary to fully evaluate that possibility.

A key idea in hippocampal long axis function is that the posterior hippocampus is well suited to supporting memory for episodic details (Brunec et al., 2018; Frank et al., 2019; Poppenk et al., 2013). Notably, however, we did not find robust relationships between any measure of hippocampal long axis specialization and episodic memory performance or its age-related change. There are several possible reasons for these null effects. First, while our episodic memory measure showed robust age-related decline, it is possible that it did not sufficiently index memory for the kinds of episodic details affected by changes to hippocampal functional specialization. Alternatively, the emotional component of the present task may have affected the relationship to hippocampal function given work suggesting a strong role of the anterior hippocampus in memory for emotional relative to neutral memoranda (Murty et al., 2010). It is also possible that functional differences across hippocampal subregions during rest are not predictive, with differences that emerge during a memory task being more indicative of an individual's memory capacity. Yet, prior work showed a relationship between within-hippocampal signals during rest and self-reported time spent in episodic simulation in a sample of young adults (Brunec et al., 2018), indicating that hippocampal subregion differences from rest can predict measures of episodic memory in some circumstances. The episodic memory measure used here was more objective, but it was not collected alongside the fMRI data. The lack of a relationship to behavior here could mean that hippocampal subregion functional differences reflect a state that can index performance in the moment but is not indicative of trait-like episodic memory abilities that are stable over longer time windows. Future work could use the task-based data from the CamCAN dataset (a movie-watching paradigm) to test whether engaging in a task is sufficient to detect a relationship between hippocampal functional specialization and age-related memory decline, even when the task is not directly related to the memory measure. In sum, while null results should be taken with caution, the present results indicate limitations in using rest-based hippocampal long axis specialization to explain age-related episodic memory decline.

While our focus in the present study was hippocampal long axis specialization across the *adult* lifespan, comparisons across the entire lifespan including child development is of future interest. Prior work has demonstrated differences in the structural and functional development of the anterior vs. posterior hippocampus (Ayoub et al., 2023; Blankenship et al., 2017; Geng et al., 2019; Gogtay et al., 2006; Zuo et al., 2025), with implications for episodic memory abilities (Riggins et al., 2016; Vijayarajah and Schlichting, 2026). Further, recent developmental work has combined multiple biological markers including hippocampal gene expression, myelination, and geometry to show extended development of hippocampal long axis gradients through adolescence (Zuo et al., 2025). Extending this approach to the full lifespan has the potential to reveal key hippocampal processes supporting both the establishment and decline of episodic memory function.

## Data Availability

The original contributions presented in the study are publicly available. This data can be found here: https://osf.io/qba8n/.
