# Supplementary material for: Adult lifespan effects on functional specialization along the hippocampal long axis
Source: Front Cognit. 2026 May 5;5:1767179. doi: 10.3389/fcogn.2026.1767179 (PMC13271125; doi:10.3389/fcogn.2026.1767179)
Supplement: Supplementary file 1 [file Data_Sheet_1.docx]

**Supplemental materials**

**Setting the threshold for the minimum number of voxels in each subregion**

The BOLD signal in individual voxels can be very noisy (Caballero-Gaudes & Reynolds, 2017; Triantafyllou et al., 2011), and a benefit of region of interest analyses is that averaging across voxels in a region can reduce noise (Poldrack, 2007). However, there is no universal standard of how many voxels are needed before the signal becomes stable. To determine a minimum number of voxels needed in each subregion for a subject to be included in the present analyses, we sought to balance signal quality (i.e., more voxels is likely to lead to better signal) with retaining as many subjects as feasible (i.e., for power in group analyses). For each subject, we computed the number of voxels included in each of the six hippocampal ROIs (tail, body, head x right and left hemisphere) after registering the ROIs into functional space. We then tested the number of subjects that would be included at minimum voxel thresholds ranging from one to twenty, assuming that every ROI would need to meet the minimum for the subject to be retained. The results are depicted in Supplemental Figure 1. We chose a minimum ROI size of 9 voxels (k > 8) because that was an inflection point where we started to lose large numbers of participants (13 subjects excluded with k > 7 threshold, 28 subjects excluded with k > 8, 59 subjects with k > 9).


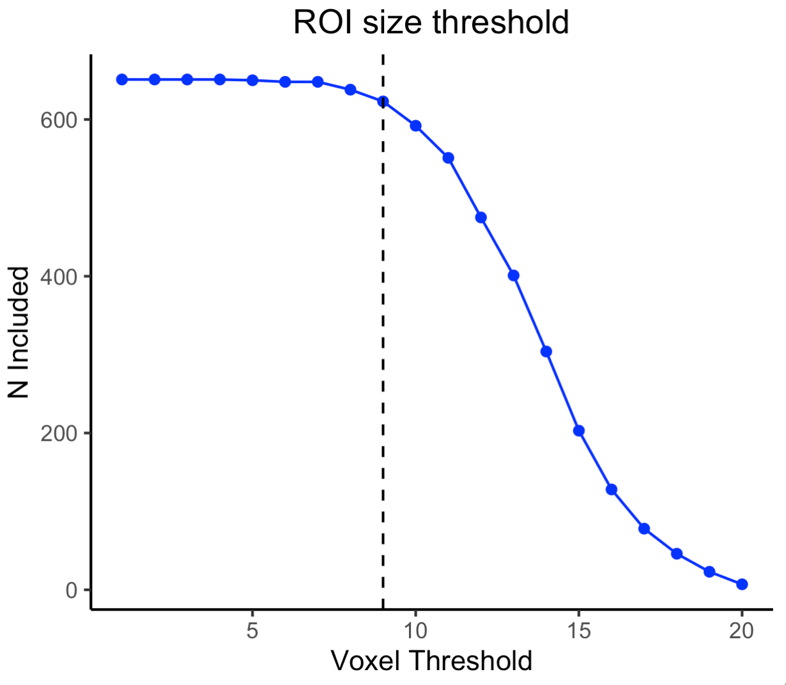


**Supplemental Figure 1***.* The number of subjects included at various thresholds for minimum ROI size. The dashed line represents the chosen threshold of k > 8 voxels (minimum k = 9).

**Temporal Signal-to-Noise Ratio (tSNR)**

Hippocampal signals derived from Freesurfer and MUSE segmentations were highly similar, but we wanted to choose the segmentation protocol that resulted in the best signal quality, even if any difference did not rise to the level of a statistically significant difference. To compare the signal quality for functional timeseries derived from Freesurfer- and MUSE-defined hippocampi, we extracted the pre-processed timeseries from the left and right hippocampi defined from each segmentation tool in each subject. We censored high motion volumes and regressed out the confound variables (FD, DVARS, translational and rotational motion, and the temporal derivatives of translational and rotational motion) from the timeseries as in the main analyses. The tSNR ws then computed on the residuals as the mean signal/standard deviation of the signal. The resulting values separated by segmentation tool and hemisphere are presented in Supplemental Figure 2. A regression model with age, segmentation tool, hemisphere, and their interactions tSNR showed no significant effects (all p’s > .13). Given that Freesurfer showed numerically higher tSNR, especially for the older subjects, we retained this segmentation method for all analyses.


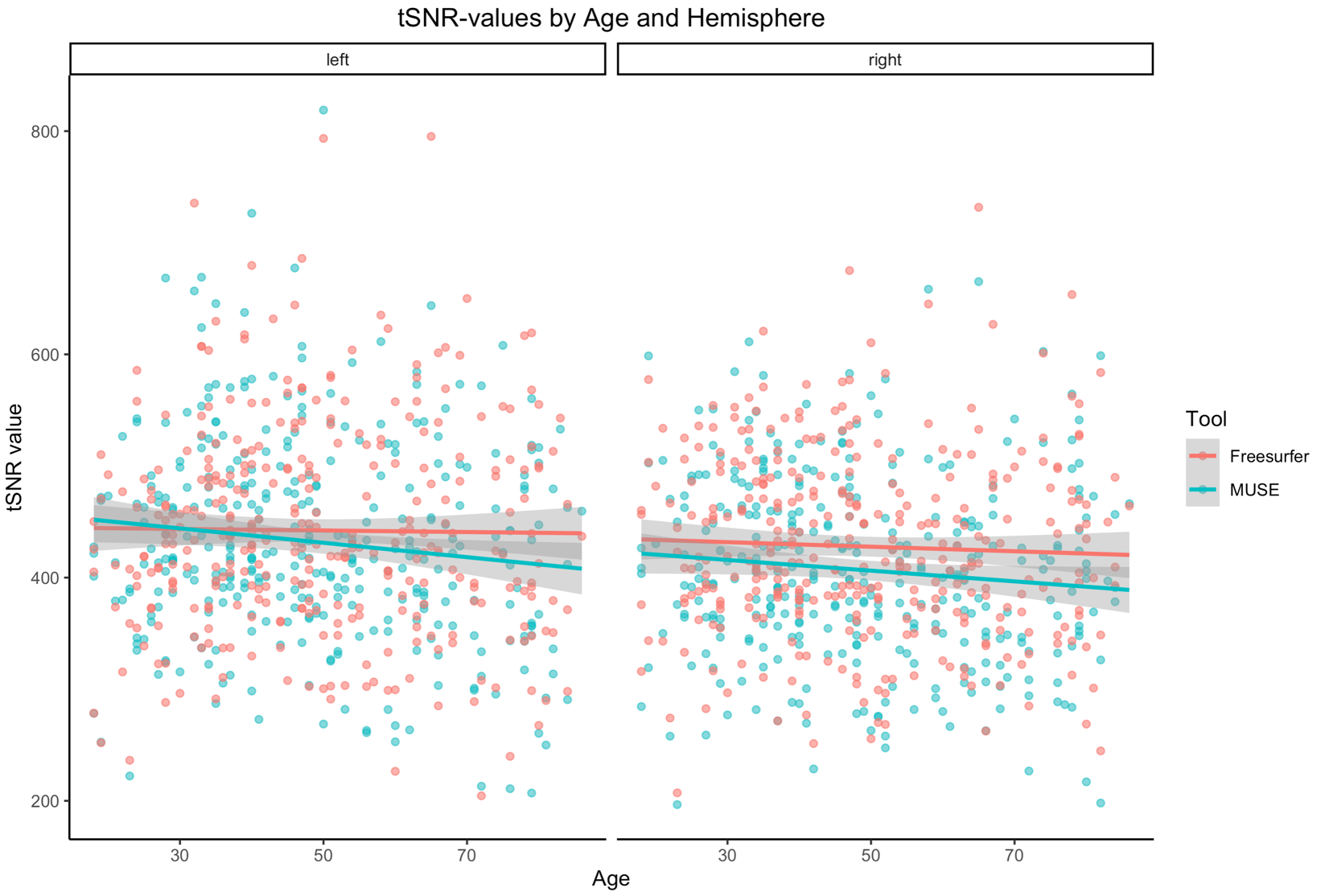


**Supplemental Figure 2.** Temporal signal-to-noise ratios (tSNR) values from preprocessed functional data. tSNR values were generated for the hippocampus as a whole from Freesurfer and MUSE segmentations separately for the right and left hemisphere. There were no significant differences in the signal quality based on software tool, age, or hemisphere, but the Freesurfer segmentations had numerically higher tSNR values and were retained for all further analyses.

| Supplemental Table 1 | | |
| --- | --- | --- |
| *List of cortical and subcortical abbreviations, full name, and Freesurfer label* | | |
| Abbreviation in Figure 4 | Full name | Freesurfer label |
| Frontal pole | Frontal pole | ctx-lh-frontalpole |
| VMPFC | Ventral medial prefrontal cortex | ctx-rh-medialorbitofrontal |
| Lateral OFC | Lateral orbitofrontal cortex | ctx-rh-lateralorbitofrontal |
| IFG (BA 44) | Inferior frontal gyrus – pars opercularis | ctx-rh-parsopercularis |
| IFG (BA 47) | Inferior frontal gyrus – pars orbitalis | ctx-rh-parsorbitalis |
| IFG (BA 45) | Inferior frontal gyrus –  pars triangularis | ctx-rh-parstriangularis |
| Rostral MFG | Rostral middle frontal cortex | ctx-rh-rostralmiddlefrontal |
| Caudal MFG | Caudal middle frontal cortex | ctx-rh-caudalmiddlefrontal |
| SFG | Superior frontal gyrus | ctx-rh-superiorfrontal |
| ACC | Rostral anterior cingulate cortex | ctx-rh-rostralanteriorcingulate |
| Mid cingulate | Caudal midcingulate cortex | ctx-rh-caudalanteriorcingulate |
| Precentral | Precentral gyrus | ctx-rh-precentral |
| Insula | Insular cortex | ctx-rh-insula |
| Temp. pole | Temporal pole | ctx-rh-temporalpole |
| Trans. Temp. | Transverse temporal gyrus | ctx-rh-transversetemporal |
| STG | Superior temporal gyrus | ctx-rh-superiortemporal |
| STS | Banks of the superior temporal sulcus | ctx-lh-bankssts |
| MTG | Middle temporal gyrus | ctx-lh-middletemporal |
| ITG | Inferior temporal gyrus | ctx-lh-inferiortemporal |
| Entorhinal | Entorhinal cortex | ctx-lh-entorhinal |
| PHG | Parahippocampal gyrus | ctx-lh-parahippocampal |
| Postcentral | Postcentral gyrus | ctx-lh-postcentral |
| Paracentral | Paracentral lobule | ctx-lh-paracentral |
| Precuneus | Precuneus | ctx-lh-precuneus |
| Sup. Parietal | Superior parietal lobule | ctx-lh-superiorparietal |
| Supramarginal | Supramarginal gyrus | ctx-lh-supramarginal |
| Inf. Parietal | Inferior parietal lobule | ctx-lh-inferiorparietal |
| PCC | Posterior cingulate cortex | ctx-lh-posteriorcingulate |
| Retrosplenial | Isthmus of cingulate gyrus | ctx-lh-isthmuscingulate |
| Fusiform | Fusiform gyrus | ctx-lh-fusiform |
| LOC | Lateral occipital cortex | ctx-lh-lateraloccipital |
| Lingual | Lingual gyrus | ctx-lh-lingual |
| Pericalcarine | Pericalcarine cortex | ctx-lh-pericalcarine |
| Cuneus | Cuneus | ctx-lh-cuneus |
| Caudate | Caudate nucleus | Left-caudate |
| Putamen | Putamen | Left-Putamen |
| Pallidum | Globus pallidus | Left-Pallidum |
| Thalamus | Thalamus | Left-Thalamus |
| Amygdala | Amygdala | Left-Amygdala |
| NAC | Nucleus accumbens area | Left-Accumbens-area |
| Ventral DC | Ventral diencephalon | Left-VentralDC |
| Cerebellum | Cerebellum | Left-Cerebellum-Cortex |

**Emotional memory task**

Participants first underwent an incidental encoding phase where they were shown a background picture for 2 seconds before an object would appear superimposed on the background. Participants were instructed to create a story linking the object to the background. The emotional component of the task came from the background pictures, which were from the International Affective Pictures Set (IAPS) (Lang et al., 1999). The background picture could depict a positive situation, a neutral situation, or a negative situation. Pairing of objects and background images was randomized such that there was no systematic semantic relationship between objects and backgrounds. Each object image and each background image was used only once. After 8 seconds, the screen would advance to the next trial. There were 120 of these trials in the study phase, with only one presentation of each object-background image pair. There was a 10-minute retention interval between the study and test phases. There were three components of each test trial, testing different aspects of memory. First, as a measure of perceptual priming, participants saw a degraded version of an object and were asked to identify it. Next, as a measure of object recognition, they saw the full, non-degraded object image and were asked whether it was old (presented in the study phase) or new, also indicating their confidence. Lastly, as a measure of associative and contextual memory, participants were asked the valence (positive, neutral, or negative) of the background image presented with the object. There were 120 trials with old, studied objects and 40 trials with new objects. In our analyses, we included the object recognition and background valence memory as indices of episodic memory.


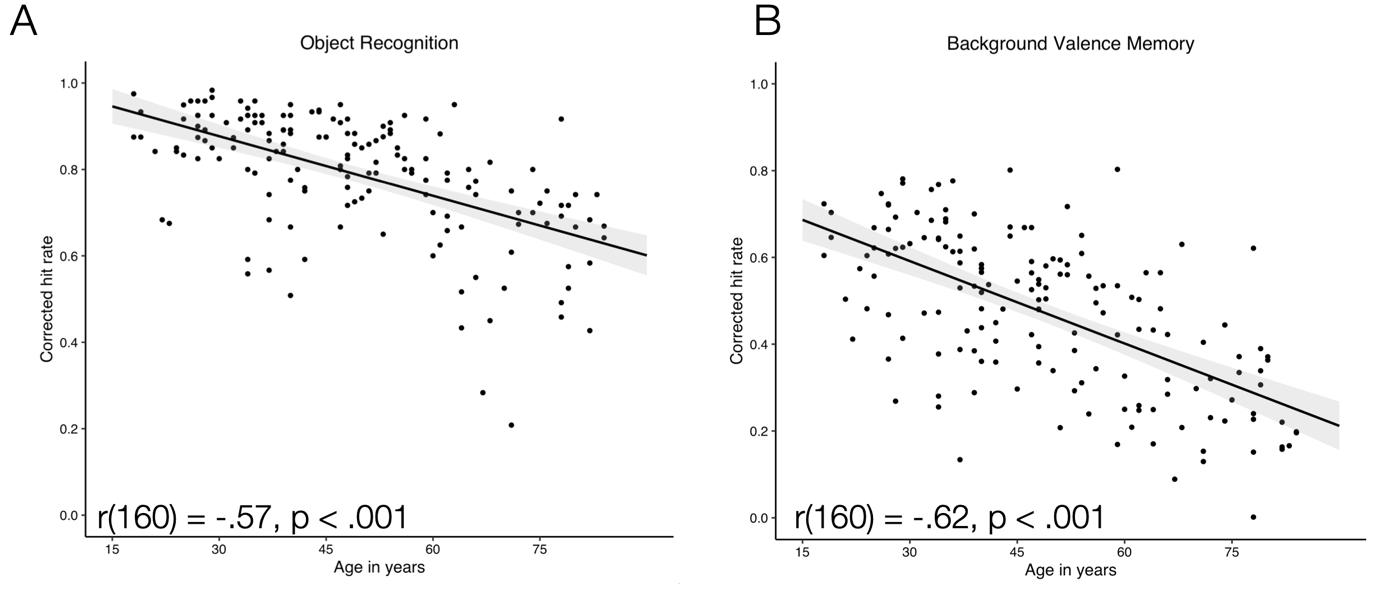


**Supplemental Figure 4. Relationship between age and episodic memory performance.**

The relationship between age (in years) and the corrected hit rate (hit rate – false alarm rate) from the (A) object recognition and (B) background picture valence tests from the episodic memory task. Pearson’s r values represent the zero-order correlation between age and the given behavioral metric.

For object recognition, we computed the average hit rate (collapsed across confidence levels) and subtracted the single false alarm rate (Supplemental Figure 3A). For the background valence memory, we averaged across the valence conditions because scores were highly correlated across subjects (Supplemental Figure 4). We computed the averaged hit rate across the three valence conditions and the average false alarm rate (e.g., a positive valence false alarm would be responding ‘positive’ to an object associated with a neutral or negative background image). We then computed the difference between the hit and false alarm rates (Supplemental Figure 3B). We then combined the object recognition and background valence measures by first z-scoring each and averaging them (Figure 2).


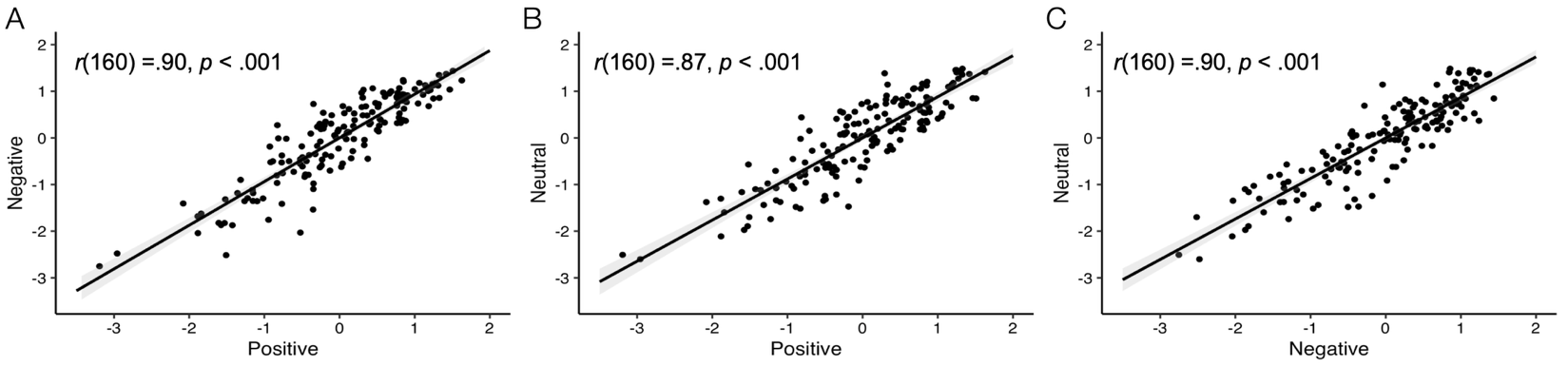


**Supplemental Figure 4. Relationships among episodic memory scores by valence.**

The relationship between the episodic memory composite scores computed separately for each valence condition. Zero order correlations between memory scores for A. positive and negative stimuli, B. positive and neutral stimuli, and C. negative and neutral stimuli.

| Supplemental Table 2 | | | |
| --- | --- | --- | --- |
| *Multiple regression relating intervoxel similarity in each hippocampal region to episodic memory performance, separated by hemisphere* | | | |
| Effect | ß | t-value | p-value |
| **Age** | **-0.034** | **-9.93** | **< .001** |
| Tail: left | 0.655 | 1.18 | .241 |
| Tail: right | -0.514 | -0.82 | .415 |
| Body: left | 0.007 | 0.01 | .993 |
| Body: right | -1.287 | -1.60 | .111 |
| Head: left | 0.681 | 0.94 | .351 |
| Head: right | 0.944 | 1.25 | .212 |
| Age x Tail: left | -0.033 | -1.02 | .308 |
| Age x Tail: right | 0.004 | 0.11 | .909 |
| Age x Body: left | 0.023 | 0.55 | .582 |
| Age x Body: right | 0.010 | 0.22 | .828 |
| Age x Head: left | -0.045 | -0.99 | .325 |
| Age x Head: right | 0.050 | 1.12 | .325 |
| **Gender (0 = F, 1 = M)** | **-0.312** | **-2.62** | **.010** |
| Hippocampal volume | 58.326 | 0.48 | .636 |
| Motion | -0.348 | -0.58 | .565 |

*Note*: bold text represents effects passing an alpha = .05 threshold.

| Supplemental Table 3 | | | |
| --- | --- | --- | --- |
| *Multiple regression relating intervoxel similarity in each hippocampal region to episodic memory performance, averaged across hemispheres* | | | |
| Effect | ß | t-value | p-value |
| **Age** | **-0.034** | **-10.09** | **< .001** |
| Tail | 0.442 | 0.75 | .457 |
| **Body** | **-1.677** | **-2.29** | **.023** |
| **Head** | **1.571** | **2.44** | **.016** |
| Age x Tail | -0.028 | -0.82 | .416 |
| Age x Body | 0.035 | 0.90 | .370 |
| Age x Head | .010 | 0.25 | .800 |
| **Gender (0 = F, 1 = M)** | **-0.311** | **-2.64** | **.009** |
| Hippocampal volume | 52.424 | 0.44 | .663 |
| Motion | -0.321 | -0.54 | .587 |

*Note*: bold text represents effects passing an alpha = .05 threshold.

| Supplemental Table 4 | | | |
| --- | --- | --- | --- |
| *Multiple regression relating the similarity connectivity profiles to episodic memory performance, separated by hemisphere* | | | |
| Effect | ß | t-value | p-value |
| **Age** | **-0.031** | **-8.78** | **< .001** |
| Tail-body: left | 0.265 | 1.85 | .066 |
| Tail-body: right | -0.058 | -0.35 | .726 |
| Body-head: left | -0.135 | -0.93 | .353 |
| Body-head: right | 0.067 | 0.38 | .704 |
| Tail-head: left | 0.171 | 1.03 | .292 |
| Tail-head: right | -0.013 | -0.07 | .946 |
| Age x Tail-body: left | 0.011 | 1.31 | .191 |
| Age x Tail-body: right | -0.005 | -0.60 | .548 |
| Age x Body-head: left | 0.011 | 1.47 | .143 |
| Age x Body-head: right | -0.009 | -1.00 | .318 |
| Age x Tail-head: left | 0.001 | 0.14 | .887 |
| Age x Tail-head: right | 0.009 | 0.94 | .348 |
| **Gender (0 = F, 1 = M)** | **-0.285** | **-2.41** | **.017** |
| Hippocampal volume | 60.997 | 0.48 | .630 |
| Motion | -0.654 | -1.12 | .264 |

*Note*: bold text represents effects passing an alpha = .05 threshold.

| Supplemental Table 5 | | | |
| --- | --- | --- | --- |
| *Multiple regression relating the similarity connectivity profiles to episodic memory performance, averaged across hemispheres* | | | |
| Effect | ß | t-value | p-value |
| **Age** | **-0.032** | **-9.30** | **< .001** |
| Tail-body | 0.195 | 1.16 | .250 |
| Body-head | -0.138 | -0.75 | .455 |
| Tail-head | 0.180 | 0.86 | .391 |
| Age x Tail-body | 0.006 | 0.70 | .483 |
| Age x Body-head | 0.003 | 0.38 | .707 |
| Age x Tail-head | 0.011 | 1.07 | .286 |
| **Gender (0 = F, 1 = M)** | **-0.298** | **-2.59** | **.011** |
| Hippocampal volume | 21.369 | 0.17 | .864 |
| Motion | -0.595 | -1.03 | .306 |

*Note*: bold text represents effects passing an alpha = .05 threshold.


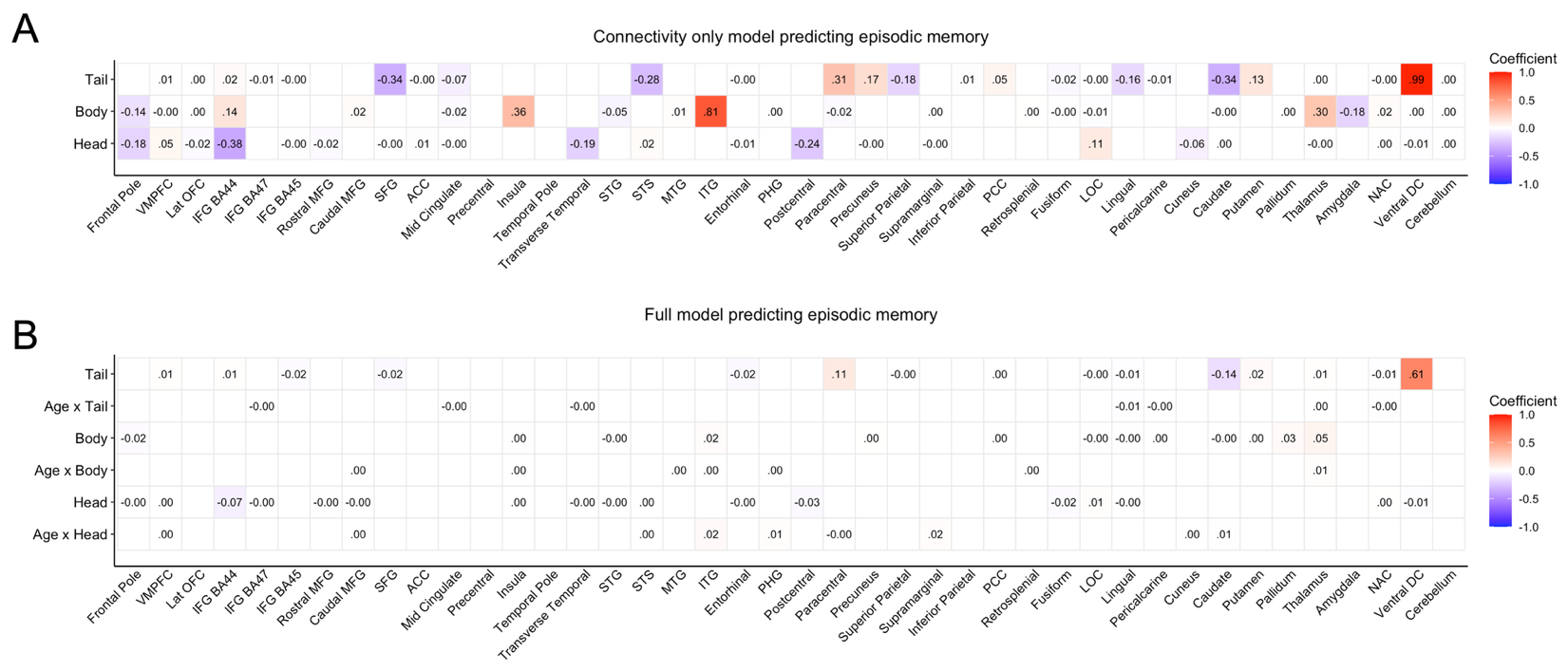


**Supplemental Figure 5. Mean coefficient estimates from the Connectivity only and Full models predicting episodic memory performance.**

(A) Coefficients from a model predicting episodic memory scores from hippocampal functional connectivity plus standard covariates without age available as a potential predictor. (B) Coefficients from a model predicting episodic memory scores from hippocampal functional connectivity plus age and age x hippocampal connectivity interactions available as potential predictors. In A-B, darker red colors represent stronger positive coefficients, darker blue colors represent stronger negative coefficients, white squares with numbers included represent coefficients that were entered into the model on at least one cross-validation fold but whose mean was near zero, and white squares with no number represent predictors that were never entered into the model across any cross-validation fold.

**References**

Caballero-Gaudes, C., & Reynolds, R. C. (2017). Methods for cleaning the BOLD fMRI signal. *NeuroImage*, *154*, 128–149. https://doi.org/10.1016/j.neuroimage.2016.12.018

Poldrack, R. A. (2007). Region of interest analysis for fMRI. *Social Cognitive and Affective Neuroscience*, *2*(1), 67–70. https://doi.org/10.1093/scan/nsm006

Triantafyllou, C., Polimeni, J. R., & Wald, L. L. (2011). Physiological noise and signal-to-noise ratio in fMRI with multi-channel array coils. *NeuroImage*, *55*(2), 597–606. https://doi.org/10.1016/j.neuroimage.2010.11.084
